# Supplementary material for: Are carbon emissions trading and green financial instruments synergistic? -Comprehensive quantitative research based on content analysis
Source: PLoS One. 2024 Mar 7;19(3):e0298601. doi: 10.1371/journal.pone.0298601 (PMC10919601; doi:10.1371/journal.pone.0298601)
Supplement: S1 Data — (ZIP) [file pone.0298601.s001.zip › Data/地级市Z_F工作报告.zip!/╝¬┴╓/░╫╔╜╩╨/└┤╫╘╣1⁄2╓┌║┼í╛┬φ┐╦╩2╛▌═°í┐_2004.docx]

各位代表：

我受白山市人民政府的委托，向大会报告工作，请予审议。

一、2003年工作基本回顾 　　2003年，是我市发展进程中十分重要而不寻常的一年。面对复杂多变的国际形势和国内突如其来的非典疫情，我们在市委的坚强领导下，团结和带领全市各族人民，牢牢抓住加快发展不放松，着力解决经济和社会生活中的突出矛盾和问题，保持了经济稳步增长和各项社会事业全面发展的良好势头，较好地完成了四届人大四次会议确定的目标任务。

——国民经济稳步增长。实现全市生产总值109亿元，比上年增长8.1%；一般预算全口径财政收入7.59亿元，增长8.4%；全社会固定资产投资达到32.5亿元，增长14.8%。城乡基础设施建设力度加大，交通建设完成投资13.1亿元，在建公路里程1180公里，靖宇至辉南地方铁路完成年度计划；城市建设总投资4亿多元，完成曲家营水库供水工程、卫国路公铁立交桥、喜丰花园等一批城市建设项目，对经济发展的拉动作用明显增强；小城镇建设取得新成效。重点技改项目完成投资6.9亿元。一批双停企业顺利启动。城乡市场转旺，消费需求进一步增强，全社会消费品零售总额完成45.6亿元，增长7.1%。

——产业结构调整积极推进。重点在做强支柱产业、培育希望产业、扶持重点企业上下功夫，工业经济发展平稳，基本遏制住了下滑势头。规模以上工业完成增加值30.1亿元，实现净利润1.48亿元，分别增长7.1%和9.2%。电力、木制品加工、煤炭、建材、矿泉水、有色金属冶炼成为拉动全市工业经济增长的主要动力，六大行业完成的总产值占规模以上工业的66.1%。农村经济结构调整力度加大，种植业结构进一步优化，产业化经营加速发展。全市共建成500亩以上的综合开发生产基地36个，新建绿色食品基地6个，我市被世界饮水组织命名为“国际矿泉城”。共完成植树造林1.4万公顷，其中退耕还林8498公顷，实现了连续24年无重大森林火灾。第三产业健康发展，旅游总收入实现2.52 亿元，房地产开发投资增长42.7%，成功举办了第二届房屋展示交易会，商贸流通等服务业取得明显成效。县域经济加快发展，综合实力有所增强。民营经济保持较好发展势头，全市规模以上民营企业达到68户。 　　——改革开放进一步加快。企业改革继续推进，全市有46户企业实施了不同形式的改革。嵩华纸业、喜丰公司、合兴实业分别完成了“一个置换、两个转变”和净资产量化改革，汽车改装厂等12户企业进行了产权出售，市酿造厂等2户企业实行了公司制改革，长白森经局等12户企业完成了资产重组，天时水泥刨花板厂破产核呆终结，轴承股份公司等5户企业依法实施了破产，西村煤矿等9户企业实施了租赁经营，通化矿务局湾沟和苇塘煤矿破产工作进入法律程序。农村税费改革取得阶段性成果。农村卫生体制改革稳步推进，各乡镇卫生院全部上划县卫生行政部门管理。事业单位改革试点基本完成。整顿市场经济秩序工作取得新成果。加大招商引资力度，全年引进国内资金12亿元，实际利用外资585万美元。对外贸易成果显著，全年完成外贸进出口总额6808万美元，增长18.6%，其中出口5714万美元，增长21.2%。

——精神文明建设和各项社会事业取得新成绩。群众性精神文明创建活动深入开展，市民文明程度不断提高。“双拥”共建活动取得新成果，长白朝鲜族自治县被命名为全国双拥模范城（县）。市外国语学校和解放中学新教学楼、临江和抚松外国语学校等一批教育基础设施相继建成，各县（市）文化馆、图书馆等项目全部启动。教育改革进一步深化，教学质量不断提高。科技项目申报和引进工作进展顺利，科技成果转化步伐加快。广播电视覆盖面不断扩大，节目制作播出质量明显提高。改造了市体育馆和滑雪场，为市区15个居民小区安装了室外健身器材。防控非典工作取得阶段性成果，有效杜绝了疫情传入，高速度、高质量地建成了市传染病医院，公共卫生体系进一步完善。稳定了低生育水平，人口自然增长率控制在3.2‰以内。 　　——人民生活继续改善。城镇居民人均可支配收入达到6556元，农民人均纯收入达到3050元，分别增长8.2%和4.7%。通化矿区采煤沉陷区综合治理项目通过国家中咨公司评估，确定白山境内治理资金8.2亿元。就业和再就业工作力度加大，全年开发就业岗位3.5万个，实现就业和再就业3万多人，劳务输出近4.5万人，城镇登记失业率控制在4%以内。社会保障体系建设继续加强，全市共筹集下岗职工基本生活保障金1967万元，为7196名下岗职工发放基本生活费1725万元，收缴社会养老保险基金2.15亿元，为6.7万离退休人员发放养老金4.4亿元，基本做到了“两个确保”；城市居民最低生活保障工作做到了动态管理、应保尽保，共为18.86万低保对象发放最低生活保障金8568万元。扶贫开发工作取得新进展，全市107个扶贫工作重点村全部落实了对口帮扶单位，全年共争取国家农村扶贫资金近2100万元。

——保持了政治安定和社会稳定。加大了对“法轮功”等邪教组织和各类刑事犯罪的打击力度，社会治安综合治理成效明显，人民群众的安全感进一步增强。顺利完成了边境防务交接，维护了边防线稳定。依法加强了民族、宗教工作。以对人民群众高度负责的态度，认真办理人民来信来访，及时妥善化解了各类社会矛盾。严格落实安全生产管理工作各项制度，特别是深入开展了以煤矿为重点的安全生产专项整治，不断加大安全生产投入和监督检查力度，探索建立长效机制，安全生产形势比较平稳，重特大事故得到有效遏制。 　　——政府自身建设进一步加强。积极推进政府职能转变，行政审批、会计核算和土地收购储备制度改革不断深化。市直33个重点部门和各县（市）区全部实行了“十公开”制度，我市被省政府评为政务公开优秀地区，市政务服务中心被列为全省政务公开高标准示范单位。继续推进了纠正部门和行业不正之风、领导干部廉洁自律、查处大案要案及治理软环境工作，开展了 “学、做、创”活动和市直机关目标责任制考核评比工作。市政府自觉接受人大的法律监督、工作监督和政协的民主监督，全年共办理人大议案、建议127件，办理政协提案90件。“四五”普法教育广泛开展。行政监察、审计监督和行政复议等工作不断加强。

一年来，统计、环保、水文、气象、人防、民兵预备役、地震监测、外事、地方志、档案、老龄、助残、关心下一代、妇女儿童等工作都为全市经济社会发展做出了积极的贡献。 　　各位代表，成绩的取得来之不易。这是在市委的领导下，全市人民开拓创新、共同奋斗的结果，凝聚着全市各级干部和各族人民群众的心血和汗水。在此，我代表市政府，向为我市改革开放和现代化建设付出辛勤劳动的全市各族人民，向给予政府工作大力支持和有效监督的人大代表、政协委员和各民主党派、工商联、人民团体、无党派人士，向为白山改革发展稳定做出积极贡献的人民解放军指战员、武警官兵、政法干警、中省属企事业单位的广大干部职工，表示崇高的敬意和衷心的感谢！

在肯定成绩的同时，我们也清醒地看到，我市经济社会生活中还存在着一些亟待解决的困难和问题，主要是：经济总量小，缺少大项目支撑，产业层次不高，经济运行质量和效益较低，发展后劲不足，地方财政收入增长乏力，收支矛盾非常突出。就业和再就业形势严峻，社会保障压力加大。软环境方面存在的问题较多，企业和群众反响强烈。政府工作还不能完全适应新形势、新任务的要求，部分干部思想不够解放，作风不够扎实，一些关系人民群众切身利益的问题没有得到很好解决，党风廉政建设还需要进一步加强。这些问题，市政府一定高度重视，采取切实有效措施认真解决。

二、全力做好2004年各项工作 　　关于今年和未来一个时期全市工作的总体要求、目标任务和工作措施，市委四届三次全会做出了全面部署，我们要认真抓好贯彻落实，务求取得实效。

2004年是实施东北老工业基地振兴战略的开局之年，做好今年的各项工作十分重要，任务也非常艰巨。市政府的工作思路是：坚持以邓小平理论和“三个代表”重要思想为指导，全面贯彻党的十六大和十六届三中全会精神，牢固树立和认真落实全面、协调、可持续的发展观，按照市委四届三次全会部署，紧紧抓住东北老工业基地调整改造的历史性机遇，以建设资源型城市发展接续产业示范基地为目标，坚持工业立市、工业强市，继续深化改革，进一步扩大开放，加快经济结构调整，突出项目建设，扩大经济总量，提高经济效益，实施全民创业，正确处理好改革发展稳定的关系，不断提高人民生活水平，加强社会主义政治文明和精神文明建设，努力实现国民经济持续协调健康发展和社会全面进步。

综合考虑各方面因素，2004年主要预期目标是：全市生产总值增长10%，财政收入增长10%，全社会固定资产投资增长15%，城镇居民人均可支配收入和农民人均纯收入分别增长8%和7%。

完成上述目标，要重点抓好以下七个方面的工作。

（一）加大结构调整力度，促进经济持续健康较快发展。

工业化水平低是我市经济结构存在的主要矛盾。要按照走新型工业化道路的要求，加大产业结构调整力度，全面提升和优化第二产业，大力发展现代农业和现代服务业，推动经济尽快步入良性发展轨道。

1．围绕工业立市，突出抓好项目建设。紧紧抓住国家扩大内需、支持东北等老工业基地调整改造的难得机遇，准确把握国家产业政策走向及投资重点，围绕支柱产业和希望产业，积极做好项目开发储备、整体包装和申报推介工作。对奥迪B6/C6轿车门内板焊接总成、华洋药业人参和西洋参茎叶深加工、农夫果园饮料系列产品深加工、葡萄籽综合开发、超微细二氧化硅、泉阳泉饮品公司野生特色资源加工等有希望列入第二批计划的项目，要进一步加大工作力度，千方百计争取列入国家计划。立足实际，超前研究、规划、论证一批产业化水平高、关联度强、符合国家产业政策的大项目、好项目，并有重点、有选择地发展一批高新技术产业，增强发展后劲。紧紧抓住落实项目资金这个关键环节，积极争取国债资金，落实承贷银行，扩大民间投资，实现重点项目投资主体多元化，把争取到的项目落到实处。要加强对项目工作的领导，进一步完善项目工作体制和运行机制，提高项目开发建设水平。

　加快骨干企业技术改造。坚持用高新技术改造传统产业，着力实施一批重大技术改造项目。一是努力抓好已建成项目的达产达效。碳化硅、镁粉、微硅粉回收、三岔子和露水河年产5万立方米均质刨花板和农夫山泉、娃哈哈矿泉水等项目，要积极开拓国内外市场，抢占市场先机，扩大市场份额，尽快实现满负荷生产。二是加快在建项目建设进度。镁合金压铸件、节水塑料器材、窑尾余热发电和靖宇德亚、双龙矿泉水等项目，要进一步落实项目法人责任制，加快建设速度，确保工程质量，年内建成投产。长白山酒业与吉源公司联合开发的长白山牌矿泉水争取尽快打入市场。完成长白山药业集团等5户医药企业的GMP认证，培育拳头产品，拓宽营销渠道，实现医药产业的快速发展。三是全力促成新项目开工建设。总投资30多亿元、年产值5.4亿元、税收1.3亿元的浑江发电公司五期60万千瓦机组扩建工程，要组织专门力量，加大攻坚力度，做好各方面协调工作，力争年内开工。嘉懋公司60万吨焦碳、金桥木业240万平方米复合地板、露水河林业局年产8万立方米防潮刨花板、抚松金隆木业复合地板三期扩建、靖宇吉强木业高档实木家具等项目，也要加快前期工作进度，积极落实开工条件。以泉阳泉牌矿泉水被评为中国名牌产品为契机，加大宣传力度，努力打造长白山矿泉水品牌优势。加强与国际知名矿泉水企业合作，积极促成与顶鑫、俊益等矿泉水生产企业的联合开发。力争用3－4年时间，使我市矿泉水生产规模达到200万吨以上。

抓好现有企业的提速增效。加强对喜丰、天成、金刚砂等20户重点企业的调度，逐户落实扭亏增盈措施，认真帮助企业解决在生产经营中遇到的困难，加大生产要素配置协调力度，确保盈利企业继续增盈，亏损企业尽快减亏。充分发挥中小企业信用担保公司作用，为中小企业提供信贷支持。

牢固树立大白山观念，全力支持中省属企业加快发展。把中省属企业作为白山经济的重要组成部分和加快发展的中坚力量，充分发挥他们的规模优势、装备优势、技术优势、管理优势和人才优势，创造良好环境，提供优质服务，支持他们稳定主业、搞活辅业、发展接续产业。对中省属企业的重点项目，各级政府要给予同地方企业平等待遇或更加优惠的政策，在立项审批、征地拆迁等各方面提供配合与服务，支持他们降低建设成本，加快工程进度。采取切实措施，鼓励和支持中省属企业收购、兼并、重组地方企业，实现低成本扩张，形成新的经济增长点。

2．加快农业产业化进程，大力发展现代农业。继续用工业化思维谋划农业发展，以市场为导向，以生态农业建设为基础，以增加农民收入为核心，努力构筑以特产业为主的山区大农业。

加快建设具有白山特色的农业产业化体系。充分发挥白山自然资源优势，把林地经济综合开发和绿色产品深加工作为农村经济发展的重点，大力发展适应农业，加快建设长白山绿色食品产业化体系、生态医药产业化体系、林木产业化体系和畜牧产业化体系。重点抓好人参、林蛙、中药材、山珍野果、食用菌、山野菜、林纸一体化、野生动物养殖等生产基地，培育壮大长白山高山越桔、大阳岔食用菌、西洋参系列产品加工、临江澳士达牛肉制品、昌隆和万隆猪场等一批有较强带动作用的龙头项目，积极推行公司加农户、公司加基地带农户的产业化运作模式，努力扩大产业规模，尽快建立起集生产、加工、销售为一体的产业体系。在传统农业方面，要紧紧抓住国家加大对粮食主产区扶持力度的有利机遇，坚持以市场为导向，进一步调整和优化种植业结构，积极扩大我市培育的长白山一号、二号优良玉米种植面积，大力发展优质高效农业，力争高效作物种植比例和优质品率分别达到45%以上，发展“千元效益田”12万亩，无公害蔬菜2万亩。

继续加强长白山生态环境建设。坚持把保护和发展以森林为母体的生态资源作为一项长期的战略任务来抓，切实强化林政管理，严厉打击各类破坏森林资源的违法犯罪活动，认真做好森林防火工作，确保实现25年无重大森林火灾。全力做好退耕还林和“三北防护林”建设，力争完成退耕还林10万亩、“三北”四期防护林4万亩，其中市区环城绿化美化1.1万亩，不断改善城乡生态环境。继续加大矿泉水资源保护工作力度，泉阳矿泉水保护区力争年内完成审批，露水河、漫江矿泉水保护区规划建设年内启动。 　　切实做好农村基础性工作。继续深化农村税费改革，严格按照国家的减免税规定，据实核减到户。坚持依法、自愿、有偿的原则，大力推进土地使用权有偿转让，不断扩大规模经营。进一步加大农田水利基本建设，完成治理水土流失面积12万亩。大力发展多种形式的农村经济合作组织，积极建设农村经济信息网络，提高农村经济的市场化程度。深入开展以推动农村富余劳动力转移、带动农民素质提高和农民致富为主要内容的“一推双带”活动。

3．以长白山旅游为重点，大力发展第三产业和现代服务业。

依托长白山品牌优势，突出原始、自然、神奇的特点，认真办好长白山野生花卉节、金秋红叶节和冰雪旅游节，全面提升白山旅游知名度。高标准启动松江河旅游经济开发区建设，深度开发龙山湖、白山湖、望天鹅、太平湖等旅游景点和探险、漂流等旅游项目，大力发展跨国旅游，着力构筑以长白山为龙头，以鸭绿江、松花江两大流域为基础的“环山、沿江、跨国”旅游经济格局。围绕吃、住、行、娱、购等旅游要素，加快星级宾馆、饭店等基础设施建设，提高白山旅游的承载能力。切实加强旅游行业管理，培养一批高层次的接待、服务、导游人才，提高从业人员素质和服务水平，不断把白山旅游产业做大做强。

努力拓展交通运输、通信、商贸流通、金融保险、信息咨询等行业的服务范围和业务领域，积极推行仓储超市、物流配送、连锁经营等现代营销方式，大力发展与人民生活密切相关的生活服务业和社区服务业，不断拓宽第三产业发展领域。进一步加强长白山山货市场、万良人参市场等专业批发市场建设，切实增强辐射带动功能。建成市物流中心，使之尽快成为白山货运的枢纽，加快推进现代服务业的发展。

4．大力发展民营经济。进一步打破体制性障碍，完善和落实鼓励民营经济发展的政策支持体系，放宽市场准入，鼓励非公有资本进入基础设施建设、公共事业以及法律法规没有禁止的其他行业和领域，在立项审批、土地使用、信贷投放、对外贸易等方面，赋予民营经济同其他各类企业平等的竞争条件。鼓励和支持民营企业通过购买、租赁、参股、控股等多种形式，参与国有、集体企业改组改造。全面实施“扶优促强”工程，加快发展资源型、科技型、外向型民营企业，努力培育一批销售收入500万元以上的民营经济大户。 　　（二）继续深化改革、扩大开放，增强经济发展动力和活力。

积极探索公有制的多种有效实现形式，推进体制和机制创新。进一步加大招商引资力度，全方位扩大开放领域，为老工业基地调整改造和发展接续产业注入生机。 　　积极推进企业改革。以建立现代产权制度为目标，以股份制、民营化为主攻方向，加快推进国有和集体企业改制。按照资产不流失、职工得安置、企业有发展、程序要合法的原则，积极稳妥，因企制宜，先易后难，进一步加大中小企业产权出售、股权转让和租赁经营等多种形式的改革力度，大力发展混合所有制经济。积极推进“一个置换、两个转变”和集体企业净资产量化改革。完成市造纸厂核呆工作，争取市纺织厂列入国家核呆计划。按照现代企业制度的要求，进一步深化企业劳动、人事、分配制度改革，创新内部管理机制。完善法人治理结构，规范改制企业股东会、董事会、监事会和经营管理者的制衡机制，保障劳动者合法权益。加快实施大企业集团战略，鼓励引导优势资产和优势资源向有实力、有发展前景的企业大户集中，重点培育一批省级“小巨人”企业。做好通化矿务局湾沟、苇塘煤矿破产工作。

以招商引资为重点，进一步扩大开放。立足我市优势资源、产业基础和骨干项目，深入开展“招商引资年”活动。确立企业在招商引资中的主体地位，充分调动方方面面的积极性，以民营资本为重点，进一步加强同国内知名企业、大企业集团、民营和外资企业的联系与交往，广泛寻求合作伙伴。加强与国际饮水组织的联系，举办好首届中国·白山国际矿泉节和国际矿泉城揭牌仪式。积极参加省组织的赴日、韩等国家的招商引资活动，集中宣传和推介白山优势项目。坚持靠政策招商、环境招商、感情招商，加强以商招商、“以外引外”和网上招商。在引进资本的同时，注重引进人才、技术和管理，进一步提高招商引资质量和水平。认真落实招商引资项目责任制，制定和实施一系列鼓励招商引资的政策措施，对有突出贡献的单位和个人给予重奖。在巩固和扩大日本、欧盟、美国、朝鲜等传统出口市场的基础上，大力开拓俄罗斯、中东和非洲市场，进一步优化出口商品结构，提高对外贸易总体效益。加快全市5个省级开发区建设，加大政策扶持力度，使之尽快成为招商引资的热点和对外开放的重要窗口。

（三）全面加强软环境建设，为经济发展创造良好的外部条件。 　　良好的发展环境，是经济社会快速发展的必要条件。要继续加大软环境整治力度，加强全社会信用体系建设，加快构筑白山经济跨越式发展的环境优势。

进一步整治经济发展软环境。深入开展改善经济发展软环境集中学习宣传教育活动，牢固树立软环境不优白山就难以发展、不整治软环境我们将错失发展机遇的忧患意识，进一步改进工作作风，增强全体公务员的服务意识，提高服务质量和效率。继续巩固集中治理乱收费、乱罚款工作成果，国家和省明令取消的、地方和部门擅自设立的收费罚款项目必须清理掉。市政府还要全面审查执收执罚单位和执法人员资格，进一步规范行政行为。认真纠正部门和行业不正之风，加大涉软案件查处力度，建立软环境案件投诉举报中心，形成直诉快查速结的工作机制。重点查处越权执法、野蛮执法、随意执法、刁难勒索、以权谋私、吃拿卡要等行为。对构成违纪的要按照市纪委、监察局《关于损害经济发展软环境行为的责任追究办法》，给予当事人纪律处分，调离岗位、辞退、直至开除公职，并追究有关领导的责任，选择典型案件公开曝光。通过一系列措施，努力解决政府权力部门化、个人化、利益化问题，逐步建立规范的行政权力运行机制，全力营造有利于经济社会发展的政策环境、服务环境、市场环境和法制环境。

加强全社会信用体系建设。各级政府要言必信、行必果，努力提高行政公信度。把信用建设作为建立现代企业制度的重要内容，规范市场主体信用行为，提高企业信用等级。强化全民诚信意识，确立社会信用准则，全面打造“信用白山”形象。深入整顿和规范市场经济秩序，严厉查处和打击制假售假、商业欺诈、合同违约等行为。加强工作的沟通与协调，不断树立和推介白山的新发展、新形象、新思维、新作风，积极协调中省直有关部门，共同关注、关心和支持白山的发展。

（四）加强基础设施建设，改善城乡环境。 　　坚持积极而为、适度超前的原则，加大工作力度，积极争取国家和省在政策、资金等方面的支持，使我市的基础设施建设有一个较大突破。

继续加快交通建设。完成公路建设投资10亿元，临江老岭隧道衬砌及引道工程、南线一级公路、白山至榆木桥子二级公路、横山至天池旅游公路以及万良至沿江通延边、十四道沟至临江通集安、靖宇至小西头通桦甸的出口公路完成年度计划。15个未通油路的乡镇全部开工，40个行政村通油路工程年内竣工。靖宇地方铁路完成年度施工任务。完成长白山支线机场前期工作，争取早日开工建设。认真做好环长白山旅游公路、沿鸭绿江边防公路、长白长松岭隧道等重点项目的前期工作。

进一步加大城市建设力度。高起点、高质量地修编《城市总体规划》，搞好江北新区设计和南山区规划。按照统一规划、综合设计、成片开发、配套建设、分步实施的原则，抓好江北新区开发，用3—5年时间建成设施完善、功能齐全的新城区。今年完成20万平方米住宅和配套设施建设。建成星泰大桥和通江桥下游钢板闸及悬索人行过江桥。完成滨江公园西段、长白山大街西段和向阳路北段建设工程，改造卫国路公铁立交桥周边环境。以儿童公园扩建改造为重点，进一步加大对金坑河的综合治理，建闸引水，改善园区及河道环境。市消防指挥中心、市区无害化垃圾处理场一期工程年内交付使用。市中心医院住院楼、紧急救援中心和医疗废物处理站，完成年度计划。加快管道燃气工程建设进度，做好市区供热三期工程和污水处理场前期工作。发动全社会力量，实施市区绿化、美化、亮化、硬化工程。改造北山公园植物配置，搞好街路和庭院绿化，对主要街路两侧和浑江沿岸的建筑，进行楼顶装饰和立面装修，安装高档灯饰，提高城市道路、小区硬覆盖率，提升城市档次和品位。不断加强社区建设，完善社区功能。强化经营城市理念，做好新城区土地收储和旧城区政策性捆绑改造工作，不断扩大基础设施冠名权、收费权、收益权范围，拓宽融资渠道，解决城建资金不足问题。进一步理顺城市管理体制，加大管理力度，提高管理水平。按照“强基、兴商、活企”的要求，重点加强县城、中心镇和旅游沿线的小城镇建设，增强辐射带动功能，加快农村城镇化进程。 　　加强水利、电力等基础设施建设。加快浑江干流和鸭绿江界河堤防建设，完成浑江干流通江桥至青沟河口段北岸江堤改造，搞好中小河流治理，完善城市防洪体系，增强防灾抗灾能力。完成大通沟供水管网改造，提高供水质量。建设36处农村饮水工程，解决部分乡镇和村屯人畜饮水难问题。完成双河电站主体工程，力争年底投产发电。续建长白横山水利枢纽，争取启动江源西北岔水利枢纽工程。完成松江河梯级双沟常规电站与抽水蓄能电站结合方案的规划设计。做好地方水电农网改造二期收尾工作。积极申报国家电源电网项目，重点抓好靖宇220千伏一次变等工程前期工作。

（五）积极推进社会主义精神文明建设，促进经济与社会全面协调发展。

切实加强精神文明建设，努力提高市民的思想道德素质、科学文化素质和健康素质，为白山建设与发展提供精神动力和智力支持。

进一步提高精神文明建设水平。认真贯彻落实《公民道德建设实施纲要》，广泛开展社会公德、职业道德和家庭美德教育。继续抓好市区科教、文体、法律、环保、卫生、计划生育“六进社区”活动，并逐步向全市延伸。继续开展“一优、两治、全民教育”，精心组织好创建文明城市、文明行业、文明村镇和军（警）民共建活动，倡导移风易俗，积极营造健康文明、奋发向上的良好氛围，全面提高城乡文明程度和市民的整体素质，努力形成加快发展、富民强市的精神动力和强大合力。 　　稳步发展各项社会事业。加快科技进步和创新，提高科技成果转化率，提升资源开发层次和水平。继续推进国民经济和社会信息化。大力拓宽科技合作领域，加强与重点高等院校、科研单位的合作，引进智力，发展自己。高度重视人才的培养、引进和使用，大力发展基础教育、职业教育和成人教育，全面提高教育质量，为白山经济建设培养大量高层次、高技能的复合型、适用型人才。认真抓好长白山文化的研究与开发，大力弘扬创业文化，宣传创业精神，营造创业氛围。继续加强县（市）文化馆、图书馆等文化基础设施建设。依法加强对文化和新闻出版市场的管理，促进文化产业健康发展。加快发展广播电视事业，努力提高广播电视节目覆盖率。不断提高竞技体育水平，广泛开展全民健身活动，增加对城市社区健身器材的投入，组织好第五届全市运动会。加快公共卫生体系建设，逐步建立健全突发公共卫生事件应急体系、疾病预防控制体系和卫生执法监督体系。加强污染防治和生态保护工作，进一步改善环境质量。切实加强人口与计划生育工作，人口自然增长率控制在5‰以内。进一步提高“双拥”工作水平。认真抓好老龄、妇女儿童和关心下一代工作。

（六）加强民主与法制建设，确保政治安定和社会稳定。

坚持依法治国和以德治国相结合，加强社会主义民主与法制建设，努力创造安定祥和的社会环境。

积极推进依法治市。自觉接受人大及其常委会的法律监督、工作监督，认真执行有关决定和决议。自觉接受人民政协的民主监督，支持其充分发挥政治协商、民主监督和参政议政职能。认真及时办理人大代表议案、建议和政协委员提案。坚持和完善政府重大事项向同级人大及其常委会报告和与政协协商制度、联系人大代表制度、议政会制度，主动加强与各民主党派、工商联、人民团体和无党派人士的联系，建立专家咨询、法律咨询和重大决策听证、公示制度，加强社会监督和舆论监督，实现决策的民主化、科学化。切实加强基层民主政治建设，充分发挥职代会、股东会、社区居委会、村委会的作用，积极推进政务公开、厂务公开和村务公开，扩大人民群众对社会事务的知情权、参与权和监督权，保障群众的合法权益。认真贯彻《行政许可法》，规范各级政府的行政行为。大力开展“四五”普法教育，提高全市公民的法律素质。切实维护民族团结，促进民族地区经济繁荣。依法加强对宗教事务的管理。 　　把维护稳定工作摆在突出位置。始终保持对各类刑事犯罪的高压严打态势，重点打击黑恶势力犯罪和杀人、爆炸、绑架、放火、投毒等严重暴力犯罪，严厉打击跨国犯罪、毁林犯罪以及盗窃、抢劫、诈骗等侵财犯罪和毒品犯罪。继续深化同“法轮功”、“实际神”邪教组织的斗争，严厉打击境内外敌对势力、敌对分子的渗透和破坏活动。深入开展矛盾纠纷排查调处工作，加快建立预警和快速反应机制。完善社会治安防控体系，预防和减少各类可防性案件的发生。以解决集体访和越级访为重点，继续坚持“分级负责、归口办理”、“谁主管、谁负责”的原则，认真落实信访工作“一岗双责”制，坚持和完善领导接待日、领导包案和律师咨询制度，严格实行责任追究制，使信访工作走上规范化、法制化轨道。变上访为下访，把矛盾解决在基层，化解在萌芽状态。继续开展以煤矿、非煤矿山和危险化学品为重点的安全专项整治，煤矿安全要突出“一通三防”，尽快建立全市安全监控调度系统，对达不到标准的矿井，一律关停整顿。坚决取缔非法矿井，严惩非法矿主。加大对交通运输、公共场所、消防安全的监督检查力度，采取有效措施消除事故隐患，从源头上控制重特大事故的发生。

（七）关注社会困难群体，努力抓好“三项工程”。

坚持立党为公、执政为民，把维护群众的根本利益放在首位，体察民意，关注民生，认真解决好群众的切身利益问题。

抓好就业和再就业工程。就业是民生之本、安国之策。要把促进就业摆在突出位置，实施积极的就业政策，把深化改革、促进发展、调整结构和扩大就业有机结合起来。大力发展市场前景好、就业岗位多的劳动密集型企业和现代服务业，最大限度地开发就业岗位。面向发达地区和国际就业市场，加强中介机构建设，拓宽劳务输出领域。坚持劳动者自主择业、市场调节就业和政府促进就业相结合的方针，引导下岗失业人员转变择业观念，鼓励自谋职业和自主创业。进一步完善就业服务体系，加强职业技术教育和劳动技能培训。认真落实税费减免、小额贷款等再就业扶持政策，对特殊群体实行再就业援助。年内实现就业和再就业2.6万人，城镇登记失业率控制在5.5%以内。

抓好社会保障工程。认真研究国家城镇社会保障体系试点政策，搞好调查研究，摸清底数，为国有企业下岗职工出中心、解除劳动关系等方面做好各项准备工作。努力提高养老基金收缴率，筹措资金4.6亿元，确保企业离退休人员养老金按时足额发放。进一步扩大养老保险覆盖面，解决好企业职工和解除劳动关系人员的参保、续保问题。不断完善失业保险制度，做好国有企业下岗职工出中心向失业保险并轨工作。建立健全城市居民最低生活保障对象的动态管理机制，确保地方财政预算资金拨付到位，加大争取国家低保资金转移支付力度，增强保障能力。进一步做好医保政策的调整和补充，解决好困难企业职工和独立人参保问题，扩大医保覆盖面，提高医疗基金收缴率。改革农村合作医疗制度，制定新型合作医疗管理办法和实施方案，科学确定参保人群和支付比例。努力解决拖欠职工和农民工工资问题。

抓好扶贫解困工程。全面落实一把手负责制，切实关心和解决困难群众生产生活问题。加快建立和完善生活补助、社会捐助和临时救助相结合的社会救助体系，多渠道筹措资金和物资，增强救助能力。做好城市扶困工作，认真开展对特困群众的普查，准确掌握动态情况，帮助他们解决生活中遇到的实际困难。稳步推进城镇特困职工社区服务站试点工作。全面开展部门包困难企业和机关干部包特困户“一帮一”活动，通过生活上扶贫，精神上扶志，能力上扶技，经济上扶业，帮助困难企业和贫困群众逐步走出困境，自主创业。继续深入开展农村扶贫工作，积极提供市场信息和政策咨询，开展科技下乡活动，努力解决老弱病残农户生产生活中的困难，帮助和引导他们尽快脱贫致富。 　　三、切实加强政府自身建设 　　新形势、新任务对政府工作和自身建设提出了更新更高的要求。我们要以建设廉洁、勤政、务实、规范、高效政府为目标，坚持“四政”方针，努力转变政府职能，进一步加强政风建设，提高行政效能和服务水平。

提高素质，建设学习型政府。大兴学习之风，树立终身学习的理念，全面提高行政领导、公务人员的理论素养和工作水平。要善于向书本学习，倡导多读书、多思考，不断提高理论素养和政策水平；要善于向他人学习，虚心借鉴发达地区先进的工作经验；要善于向实践学习，问计于群众，求知于实践，及时总结推广群众创造的新鲜经验。坚持理论联系实际，围绕关系全市经济社会发展的重大问题，开展经常性的专题学习与研究，做到学有所获，学以致用，努力提高从政治上把握大局、从经济上驾驭全局、从理论和实践结合上解决现实问题的能力，实现自我完善和自我超越，全面提高各级政府公务员的综合素质和行政水平。 　　与时俱进，建设创新型政府。适应老工业基地调整改造和白山“二次创业”的需要，牢固树立加快发展的紧迫感和责任感，打破常规，克服惰性，在各项工作中保持敢闯、敢试、敢干的勇气，大胆改革创新，锐意开拓进取，把执行政策的原则性与灵活性结合起来，把上级要求与本地实际结合起来，创造性地开展工作。要敢为人先，敢想前人未想过的事，敢走前人未走过的路；要允许失误、允许挫折、允许特事特办，不允许回避矛盾、畏首畏尾、停滞不前，不做“维持会”，不当“太平官”，争做解放思想的楷模、勇于创新的楷模、与时俱进的楷模。

转变职能，建设服务型政府。强化政府社会管理和公共服务职能，推进公共服务体系建设，切实把政府管理职能转到主要为市场主体服务和创造良好发展环境上来。继续深化行政审批制度改革，在抓好第三批项目进厅的同时，努力解决进厅部门授权不充分等突出问题，强化窗口的审批服务职能，适当增加便民服务项目，启动网上审批试点，抓好外来投资项目服务中心工作，加快建设各县（市）区政务大厅。积极推进财政管理体制改革，尽快在全市推行会计集中核算制度，逐步探索将部门所属全额拨款事业单位的财务纳入核算中心集中管理。做好政府采购管理和执行机构分设工作，继续扩大政府采购范围和规模，从定点采购向批量招标采购发展。稳步推进事业单位改革和人事制度改革。大力实施“阳光政务”，拓展政务公开的内容和形式，加强市长公开电话工作，建成市政府门户网站、县（市）区政府网站和部分部门网站，建立和完善新闻发布会等政府信息公开制度，形成社会监督机制。

改进作风，建设务实型政府。空谈误国，实干兴邦。当前，全市大政方针已定，我们要以时不我待、只争朝夕的精神抓好落实，以埋头苦干、务求实效的态度推进落实，以行之有效的责任制度和督查机制确保落实，以实实在在、群众认可的政绩检验落实。要重实情，真正放下架子，伏下身子，用更多的时间和精力深入基层调查研究，看看到底是哪些问题制约了白山的发展，找找我们的工作还存在哪些差距，问问老百姓还有哪些实际困难需要解决。要干实事，发扬自加压力、迎难而上的精神，抓住主要矛盾，在重点工作、关键问题上勇于挑重担，善于解难题。要求实效，每个经济指标，每项工作任务，每个办事环节，对人民群众的每项承诺，都要有始有终，抓出实际效果，经得起实践、群众和历史的检验。按照省委统一部署，认真开展好“学、做、创”活动，进一步完善市直机关工作目标责任制，把树立务实的机关作风和正确的政绩观作为加强机关建设的重要内容，健全奖惩机制，使勤政为民、求真务实的干部得到褒奖，好大喜功、弄虚作假的干部受到惩戒。

从严治政，建设廉洁型政府。各级干部要认真履行党风廉政建设“一岗双责”制，教育政府公务员时刻警省自身，常修为政之德，常思贪欲之害，常怀律己之心，耐得住清贫，经得住诱惑，守得住节操。进一步规范用权、理财、管人行为，全面推行领导干部经济责任审计制度，完善监督制约机制，把法律监督、纪律监督、民主监督、舆论监督和群众监督结合起来，使各种形式的监督形成合力。要加大违法违纪案件查处力度，对滥用职权、以权谋私、贪污受贿等问题要一查到底，严肃处理，决不手软，以廉政建设和反腐败斗争的实际成果取信于民。

各位代表，新的一年各项任务非常艰巨，责任重大。我们将在市委的正确领导下，紧紧依靠133万白山人民，积极取得社会各界的广泛支持，凝聚人心，坚定信念，励精图治，开拓进取，为圆满完成今年的各项任务而努力奋斗！
